# Visual Field Testing Frequency and Associations in Children With Glaucoma

**DOI:** 10.1097/IJG.0000000000002406

**Published:** 2024-04-23

**Authors:** Natan Hekmatjah, Anika Kumar, Yinxi Yu, David N. Younessi, Ying Han, Gui-Shuang Ying, Julius T. Oatts

**Affiliations:** * University of California San Francisco School of Medicine, San Francisco, CA; § Department of Ophthalmology, University of California San Francisco, San Francisco, CA; ∥ The Francis I. Proctor Foundation for Research in Ophthalmology, San Francisco, CA; † Center for Preventive Ophthalmology and Biostatistics, Scheie Eye Institute, Perelman School of Medicine at the University of Pennsylvania, Philadelphia, PA; ‡ Northwestern University Feinberg School of Medicine, Chicago, IL

**Keywords:** perimetry, visual field testing, childhood glaucoma, glaucoma

## Abstract

**Précis:**

Children with glaucoma had an average of 1.3 visual field tests per year. Self-reported black and multiracial patients had lower visual field testing rates, whereas older children with better visual acuity had more frequent testing.

**Purpose:**

To evaluate frequency of visual field (VF) testing in children with glaucoma and identify characteristics associated with VF frequency.

**Methods:**

A retrospective cohort study of 82 children 6–18 years of age with glaucoma seen between August 2018 and May 2023. Patients were divided into those who had ≥1 VF test (303 VF tests of 61 children) and 0 VFs (21 children). Eyes were excluded if best corrected visual acuity (BCVA) was counting fingers or worse. Characteristics obtained included age, self-reported race and ethnicity, sex, primary language, glaucoma diagnosis, distance to provider, office visit frequency, follow-up compliance, insurance type, and BCVA. The main outcome measure was VF testing frequency.

**Results:**

Among children with ≥1 VF test, mean age at first VF was 11.8±2.8 years, mean number of VF/year was 1.3±0.8, and 44.9% of all VFs were reliable. Thirty nine percent of patients underwent <1 VF/year, 45.9% ≥1 to <2 VFs/year, and 14.8% ≥2 VF/year. Children who were black or multiracial had significantly lower VF testing frequency [estimated difference (ED) −1.2 (95% CI, −2.0 to −0.4, *P*=0.002) and ED −1.3 (95% CI, −2.2 to −0.3, *P*=0.008), respectively]. Better visual acuity and greater office visit frequency were significantly associated with higher VF testing frequency [ED 0.052 (95% CI, 0.001–0.103, *P*=0.045) and ED 0.2 (95% CI, 0.1–0.3, *P*<0.001), respectively].

**Conclusions:**

Most children had between 1 and 2 VF/year, although less than half of all VFs were reliable. Ophthalmologists should consider barriers to care in glaucoma monitoring.

Glaucoma, the leading cause of irreversible blindness worldwide,^1^ requires multimodal monitoring which includes intraocular pressure (IOP) measurement, assessment of the optic nerve, and visual field (VF) testing.^2^ Timely identification, management, and treatment of glaucoma progression is critical to prevent vision loss and blindness. The American Academy of Ophthalmology recommends VF testing at least annually in adults with glaucoma,^2^ with more frequent testing shown to better track glaucoma progression.^3^ Current frequency of VF testing in adults varies and is below the recommended guidelines. A recent study evaluating 380,029 patients with glaucoma from 2008 to 2017 found that >75% of patients received <1 VFs/year, with a median of 0.63 VFs/year.^4^ Another study from England found an average of 0.7 VFs/year in adult patients with glaucoma.^5^

Unlike for adults, there are no formal recommendations for VF testing frequency in children. Although glaucoma in children is uncommon,^6^ it is responsible for 5% of pediatric blindness worldwide,^7^ and there is little information regarding VF testing frequency in children. Therefore, understanding the rate of VF testing frequency in children and its associations is important to form evidence-based guidelines for VF testing in this unique population. The goal of this study was to evaluate the VF testing frequency in children with glaucoma and identify clinical and sociodemographic characteristics associated with VF testing frequency.

## MATERIALS AND METHODS

### Participants and Study Design

This was a single-center retrospective chart review approved by the University of California, San Francisco (UCSF) Institutional Review Board, which adhered to the tenets of the Declaration of Helsinki and the Health Insurance Portability and Accountability Act.

The study population included children 6–18 years of age with a diagnosis of glaucoma seen at the UCSF Pediatric Ophthalmology Glaucoma Clinic between August 2018 and May 2023. Patients were divided into 2 groups: those who had ≥1 VF test and those that had 0 VF tests. Patients were excluded if they were <6 or >18 years of age at first VF, VFs were excluded after age 18 years, and eyes were excluded if best corrected visual acuity (BCVA) was counting fingers or worse. Baseline characteristics were extracted including age, self-reported race and ethnicity, sex, primary language, glaucoma diagnosis, distance to provider (calculated using Google Maps based on patient’s address at the first visit), office visit frequency (during the study period), follow-up compliance (during the study period), insurance type, and BCVA. BCVA was converted from Snellen acuity into logarithm of minimum angle of resolution (logMAR). VFs were performed using the Humphrey Field Analyzer (Carl Zeiss Meditec) with 24-2 Swedish Interactive Thresholding Algorithm (SITA) Standard or SITA Fast testing algorithms. VF data were collected including visual field index (VFI), mean deviation (MD), and reliability metrics (false positives, false negatives, and fixation losses). Reliability was defined based on manufacturer guidelines: ≤33% false positives, ≤33% false negatives, and ≤20% fixation losses.^8^

The main outcome measure was VF testing frequency, determined by the number of VFs during the period from a patient’s first VF and the study end date (May 2023). Office visit frequency was also determined in a similar manner measuring the number of office visits during the period from a patient’s first office visit and the study end date. Compliance was measured using an average compliance score calculated based on a patient’s adherence to recommended follow-up visits across all office visits during the study period. The score was calculated as follows: follow-up ≤90 days of recommended received a score of 1, follow-up >90 and ≤180 days of recommended received a score of 0.5, and follow-up >180 days of recommended received a score of 0.

### Statistical Analysis

A two-sample *t* test or ANOVA was used for comparison of means and the chi-square test or Fisher exact test was used for comparison of percentages. Logistic regression models were used to assess baseline factors associated with patients having ≥1 VF test or not. Among patients with ≥1 VF test, linear regression models were used to determine risk factors associated with VF testing frequency (ie, number of VF tests/year), and the difference and its 95% confidence intervals (CIs) between levels of a risk factors were estimated from regression models. Factors with *a P* value of ≤0.2 from univariable regression models were selected as potential risk factors for inclusion into multivariable models. The multivariable models went through forward variable selection, and the final multivariable model only kept factors associated with *a P* value ≤0.05. Statistical significance was set at a two-sided *P* value of <0.05. All statistical analyses were performed in SAS v9.4 (SAS Institute, Inc.).

## RESULTS

### Analysis for Comparing Children With Versus Without VF Tests

The study included 303 VFs from 61 patients with ≥1 VF test and 21 patients without any VF tests. Patient characteristics are shown in Table 1. Among those with ≥1 VF test, mean age at first VF was 11.8±2.8 years, 57.4% were female, 75.4% spoke English as their primary language, 62.3% had public insurance, and mean logMAR acuity in the better eye was 0.3±0.4. Self-reported race was as follows: white (18.0%), Asian (13.1%), black (8.2%), multiracial (4.9%), or other/declined to state (55.7%). Self-reported ethnicity included Hispanic or Latino (44.3%), not Hispanic or Latino (52.5%), or unknown (3.3%). Among those without VF tests, mean age at first office visit was 9.8±4.0 years, 38.1% were female, 66.7% spoke English as their primary language, 71.4% had public insurance, and mean logMAR acuity in the better eye was 0.7±0.5. Self-reported race included white (4.8%), Asian (14.3%), black (19.0%), multiracial (0%), or other/declined to state (61.9%). Self-reported ethnicity included Hispanic or Latino (57.1%), not Hispanic or Latino (38.1%), or unknown (4.8%). Comparing those who underwent VF testing to those that did not, there were significant differences in age (*P*=0.003), BCVA (*P*<0.001), and glaucoma diagnosis (*P*=0.02). Those without VF tests were younger (9.8 vs. 11.8 y), and were more likely to have poor vision, glaucoma following cataract surgery, and glaucoma associated with nonacquired systemic disease. There were no statistically significant differences in self-reported race or ethnicity, insurance status, or distance to provider between the 2 groups (*P*≥0.32). In multivariable analysis (Supplemental Table 2, Supplemental Digital Content 1, http://links.lww.com/IJG/A890), age and BCVA in better eye were significantly associated with having ≥1 VF test. Specifically, for every 1 year increase in age, there was a 28% increase in odds of having ≥1 VF test (*P*=0.01) and for every 0.1 improvement in logMAR BCVA, there was a 27% increase in odds of having ≥1 VF test (*P*<0.001).

**TABLE 1:** Demographics of Patients With and Without Visual Field Tests

	0 Visual field test (N=21)	≥1 Visual field test (N=61)	*P*
Age (y) at first office visit or first visual field test*			**0.003**
Mean (SD)	9.8 (4.0)	11.8 (2.8)	
Median (range)	8.1 (6.1–18.2)	11.4 (6.8–18.7)	
Q1, Q3	6.9, 10.1	9.7, 13.7	
Self-reported race			0.32
White	1 (4.8%)	11 (18.0%)	
Asian	3 (14.3%)	8 (13.1%)	
Black	4 (19.0%)	5 (8.2%)	
Multiracial	0 (0.0%)	3 (4.9%)	
Other or declined to state	13 (61.9%)	34 (55.7%)	
Ethnicity			0.52
Hispanic or Latino	12 (57.1%)	27 (44.3%)	
Not Hispanic or Latino	8 (38.1%)	32 (52.5%)	
Unknown	1 (4.8%)	2 (3.3%)	
Sex (% male)	13 (61.9%)	26 (42.6%)	0.13
Primary language			0.20
English	14 (66.7%)	46 (75.4%)	
Spanish	7 (33.3%)	11 (18.0%)	
Other	0 (0.0%)	4 (6.6%)	
Glaucoma diagnosis			**0.02**
Primary congenital glaucoma	2 (9.5%)	15 (24.6%)	
Juvenile open angle glaucoma	0 (0.0%)	5 (8.2%)	
Associated with nonacquired ocular anomalies	4 (19.0%)	11 (18.0%)	
Associated with nonacquired systemic disease	5 (23.8%)	3 (4.9%)	
Associated with acquired conditions	1 (4.8%)	13 (21.3%)	
Glaucoma following cataract surgery	9 (42.9%)	14 (23.0%)	
Distance to provider (miles)			0.74
0–25	6 (28.6%)	24 (39.3%)	
25–50	5 (23.8%)	15 (24.6%)	
50–200	7 (33.3%)	17 (27.9%)	
>200	3 (14.3%)	5 (8.2%)	
Insurance type			0.59
No insurance	0 (0.0%)	2 (3.3%)	
Public insurance	15 (71.4%)	38 (62.3%)	
Private insurance	6 (28.6%)	21 (34.4%)	
logMAR visual acuity at first office visit or first visual field test,† better eye			**<0.001**
Mean (SD)	0.7 (0.5)	0.3 (0.4)	
Median (range)	0.8 (0.0–1.7)	0.2 (−0.1 to 1.3)	
Q1, Q3	0.4, 1.0	0.0, 0.4	
logMAR visual acuity at first office visit or first visual field test,† worse eye			**0.006**
Mean (SD)	1.0 (0.5)	0.6 (0.5)	
Median (range)	1.0 (0.0–2.0)	0.5 (0.0–1.8)	
Q1, Q3	0.8, 1.3	0.2, 1.1	

Bolded *P* values are statistically significant.

* Age at first office visit for those with 0 visual field tests; age at first visual field test for those with ≥1 visual field tests.

† logMAR visual acuity at first office visit for those with 0 visual field tests; logMAR visual acuity at first visual field test for those with ≥1 visual fields tests.

### Analysis for VF Test Frequency and Associated Factors

VF testing frequency, VF characteristics, office visit frequency, and compliance score data are shown in Table 2 for those with ≥1 VF test. The mean follow-up between first and last VF was 2.6±1.5 years. The mean number of VF/year was 1.3±0.8, with 39.3% of patients having <1 VF/year, 45.9% ≥1 to <2 VFs/year, and 14.8% ≥2 VFs/year. Among the total of 303 VF tests, 44.9% were reliable. In the better eye (based on BCVA), mean VFI was 90±20%, and MD was -6.3±6.4. The mean number of office visits per year was 2.6±2.0, and the mean compliance score was 0.8±0.2, meaning the majority of patients had good compliance and followed up within 90 days of the recommended visit. Patient characteristics stratified by VF testing frequency are shown in Supplemental Table 4, Supplemental Digital Content 2, http://links.lww.com/IJG/A891. There were several significant differences among groups including self-reported race (*P*=0.04), BCVA in the better eye (*P*=0.03), VF follow-up length (*P*<0.001), office visit frequency (*P*<0.001), and mean compliance score (*P*=0.03). Those with higher VF testing frequency were less likely to self-report black race, had better vision, shorter follow-up length, more frequent office visits, and higher compliance.

**TABLE 2:** Visual Field Frequency and Characteristics

		Total (N=61)
First VF VFI (percentage)	Better eye	Worse eye
Mean (SD)	90 (20)	70 (30)
Median (range)	100 (20–100)	80 (0–100)
Q1, Q3	90, 100	60, 100
First VF MD	Better eye	Worse eye
Mean (SD)	−6.3 (6.4)	−11.8 (8.4)
Median (range)	−4.9 (−28.3 to 4.10)	−9.7 (−32.9 to 0.2)
Q1, Q3	−8.1, −2.1	−18.2, −5.6
VF follow-up length (y)		
Mean (SD)		2.6 (1.5)
Median (range)		2.7 (0.2–4.8)
Q1, Q3		1.2, 4.1
Number of VFs (patients)		
1		18 (29.5%)
2		13 (21.3%)
3		14 (23.0%)
4		5 (8.2%)
5		7 (11.5%)
6		3 (4.9%)
7		1 (1.6%)
No. VF per year		
Mean (SD)		1.3 (0.8)
Median (range)		1.1 (0.3–4.2)
Q1, Q3		0.7, 1.5
VF testing frequency (per year)		
<1		24 (39.3%)
≥1 to <2		28 (45.9%)
≥2		9 (14.8%)
No. office visits per year		
Mean (SD)		2.6 (2.0)
Median (range)		2.0 (0.2–9.4)
Q1, Q3		1.3, 3.0
Compliance score		
Mean (SD)		0.8 (0.2)
Median (range)		0.9 (0.0–1.0)
Q1, Q3		0.8, 1.0

MD indicates mean deviation; VF, visual field; VFI, visual field index.

The results of the univariable analysis for factors associated with VF frequency are shown in Table 3. Self-reported race (*P*=0.01), logMAR visual acuity in the better eye (*P*=0.045), and office visit frequency (*P*<0.001) were all significantly associated with VF testing frequency. Specifically, compared with Asian patients, black and multiracial patients had significantly lower VF testing frequency [estimated difference −1.2 (95% CI, −2.0 to −0.4, *P*=0.002) and estimated difference −1.3 (95% CI, −2.2 to −0.3, *P*=0.008), respectively]. Better visual acuity and greater office visit frequency were significantly associated with higher rates of VF frequency [estimated difference 0.052 (95% CI, 0.001–0.103, *P*=0.045) and estimated difference 0.2 (95% CI, 0.1–0.3, *P*<0.001), respectively]. In addition, speaking a language other than English or Spanish was associated with higher VF testing frequency [estimated difference 0.8 (95% CI, 0.1–1.6, *P*=0.04)] compared with patients that spoke English, although this was a small subgroup of patients. Although not reaching statistical significance, there was a trend toward higher VF frequency being associated with a higher compliance score [estimated difference 0.7 (95% CI, −0.04 to 1.4, *P*=0.07)]. Black patients had significantly lower compliance scores compared with all other groups (Supplemental Table 6, Supplemental Digital Content 3, http://links.lww.com/IJG/A892). No factors were found to be significant in the multivariable models for factors associated with VF testing frequency.

**TABLE 3:** Univariable Linear Regression for Factors Associated With VF Testing Frequency

	No. patients	Mean VF per year (SE)	Estimated difference in VF/year (95% CI)	*P*
Age at first VF (per year increase)	61		−0.01 (−0.1 to 0.1)	0.81
Self-reported race				**0.01**
Asian	8	1.9 (0.3)	Reference	
White	11	1.3 (0.2)	−0.6 (−1.2 to 0.1)	0.08
Black	5	0.6 (0.3)	−1.2 (−2.0 to −0.4)	**0.002**
Multiracial	3	0.6 (0.4)	−1.3 (−2.2 to −0.3)	**0.008**
Other or declined to state	34	1.3 (0.1)	−0.5 (−1.1 to 0.01)	0.06
Ethnicity				0.55
Hispanic or Latino	27	1.3 (0.2)	Reference	0.80
Not Hispanic or Latino	32	1.3 (0.1)	0.1 (−0.3 to 0.4)	0.31
Unknown	2	0.7 (0.5)	−0.6 (−1.7 to 0.5)	
Sex				0.78
Female	35	1.2 (0.1)	Reference	
Male	26	1.1 (0.2)	−0.1 (−0.4 to 0.3)	0.78
Primary language				0.07
English	46	1.2 (0.1)	Reference	
Spanish	11	1.5 (0.2)	0.4 (−0.1 to 0.8)	0.16
Other	4	2.0 (0.4)	0.8 (0.1 to 1.6)	**0.04**
Glaucoma diagnosis				0.69
Primary congenital glaucoma	15	1.2 (0.2)	Reference	
Juvenile open angle glaucoma	5	1.0 (0.3)	−0.2 (−1.0 to 0.6)	0.61
Associated with nonacquired ocular anomalies	11	1.4 (0.2)	0.1 (−0.4 to 0.7)	0.63
Associated with nonacquired systemic disease or syndrome	3	1.0 (0.4)	−0.3 (−1.2 to 0.7)	0.59
Associated with acquired conditions	13	1.5 (0.2)	0.3 (−0.2 to 0.9)	0.27
Glaucoma following cataract surgery	14	1.2 (0.2)	−0.04 (−0.6 to 0.5)	0.90
Distance to provider (miles)				0.53
0-25	24	1.4 (0.2)	Reference	
25-50	15	1.1 (0.2)	−0.3 (−0.8 to 0.2)	0.25
50-200	17	1.4 (0.2)	0.1 (−0.4 to 0.5)	0.83
>200	5	1.1 (0.3)	−0.3 (−1.0 to 0.4)	0.40
Insurance type				0.23
No insurance	2	2.0 (0.5)	Reference	
Public insurance	38	1.2 (0.1)	−0.8 (−1.9 to 0.3)	0.15
Private insurance	21	1.4 (0.2)	−0.6 (−1.6 to 0.5)	0.32
logMAR visual acuity,* better eye (per 0.1 unit decrease)	61		0.052 (0.001 to 0.103)	**0.045**
logMAR visual acuity,* worse eye (per 0.1 unit increase)	61		0.024 (−0.014 to 0.062)	0.22
VFI,* better eye (per 1% increase)	59		0.4 (−0.8 to 1.5)	0.53
VFI,* worse eye (per 1% increase)	59		−0.1 (−0.9 to 0.7)	0.84
MD,* better eye (per 1 dB increase)	60		0.02 (−0.01 to 0.1)	0.27
MD,* worse eye (per 1 dB increase)	60		0.00 (−0.02 to 0.03)	0.73
Office visit frequency (per 1 visit/year increase)	61		0.2 (0.1 to 0.3)	**<0.001**
Compliance score (per 1 unit compliance increase)	57		0.7 (−0.0 to 1.4)	0.07

Bolded *P* values are statistically significant.

* logMAR, VFI, and MD taken from first visual field test.

CI indicates confidence interval; MD, mean deviation; VF, visual field; VFI, visual field index.

## DISCUSSION

In our cohort of children with glaucoma, the average number of VF tests/year was 1.3. Although most children (45.9%) had 1–2 VF/year, nearly 40% of patients received <1 VF/year. Black and multiracial children had significantly lower VF testing frequency. Better visual acuity and more frequent office visits were associated with higher VF testing frequency. Children who did not have VF testing were younger, had worse visual acuity, and were more likely to have glaucoma following cataract surgery or glaucoma associated with nonacquired systemic disease.

There is limited information on VF testing frequency in children, though several studies have evaluated this metric in adults, reporting between 0.63 and 0.7 VF/year in adults with glaucoma.^4,5^ The mean number of VF/year in our study was 1.3, higher than reports in adults. One potential reason for this is the high rate of unreliable VF tests in children. Several studies have demonstrated low rates of reliability in childhood VF testing, and only 44.9% of the VF in our study were reliable, similar to prior reports.^9^ In this case, the higher number of VF per year may be more related to attempting to obtain a reliable field than tracking glaucomatous visual field progression. In addition, there are currently no formal recommendations for VF testing frequency in children with glaucoma. Nearly 40% of our patients received <1 VF/year, which would be considered inadequate based on the American Academy of Ophthalmology recommendations for adults with glaucoma.^2^ Several unique considerations exist for children with glaucoma which may ultimately limit the applicability of adult guidelines to this population. Testing reliability is relatively low and generally improves with age.^10^ In addition, decreased vision from amblyopia may limit a child’s ability to complete reliable VF testing.

Our analysis revealed differences in VF testing frequency based on self-reported race, with black and multiracial children having significantly lower VF testing frequency. It is well documented that black patients, as well as other historically marginalized groups, experience discrimination in the healthcare setting and often undergo recommended testing less frequently.^11^ For example, adult black patients have been found to have a lower likelihood of receiving preventative procedures and mental health services compared with their white counterparts during primary care visits.^12^ In children, those from disadvantaged backgrounds undergo vision screening less often than white, non-Hispanic children.^13^ We found significantly lower compliance in self-reported black patients. Failure to adhere to recommended follow-up could account for the lower VF testing frequency observed in this group. Addressing barriers to follow-up in this group could help address disparities in VF testing frequency. Interestingly, we found no relationship between insurance type or distance to provider with VF testing frequency and future studies could explore these associations.

As expected, children with more frequent office visits had higher VF testing frequency. In primary care, research has shown that more visits per year are associated with improved long-term health outcomes, decreased healthcare costs, and higher utilization of evidence-based care interventions.^14^ Similarly, this association with higher VF testing frequency suggests that a proactive approach to follow-up may enhance VF surveillance. Although not statistically significant, there was a trend toward higher VF testing frequency in patients who were more compliant with recommended follow-up. Follow-up plays an important role in patient care, and prior studies have shown that higher follow-up adherence leads to improved outcomes for adults with glaucoma.^15,16^ This highlights the potential importance of a patient’s adherence to their glaucoma management and VF testing and how addressing follow-up adherence has the potential to also address VF testing frequency.

We compared demographic data for children who underwent VF testing with those who did not. Visual acuity was worse in the group with no VF testing. This could be a reflection of vision loss from advanced disease not compatible with reliable visual field testing or also could be a reflection of some developmental difference that prevented children from participating in both visual acuity testing and perimetry. We also found that children without VF testing were younger. This potentially reflects the difficulty for younger children to complete VF testing, as well as the unreliability of VF tests in younger patients, demonstrating the better use of VF testing as children get older. Every 1-year increase in age was associated with a 28% increased odds of having ≥1 VF test and every 0.1 logMAR improvement in BCVA was associated with a 27% increased odds. Type of glaucoma was different between groups and those that did not have VF testing were more likely to have glaucoma associated with nonacquired systemic disease or glaucoma following cataract surgery. For the former diagnosis, systemic disease in children is often associated with developmental or neurologic pathology, which may preclude VF testing. For the latter, overall visual acuity was worse, which may explain the difference in VF testing frequency. It may be useful for clinicians to document the specific reason for not performing VF testing to better understand these associations.

Our study has several limitations related to its retrospective study design. Furthermore, the sample size is relatively small but acceptable given the rarity of childhood glaucoma. In addition, there were 3 self-reported multiracial patients but several factors in the multivariable logistic regression (Supplemental Table 2, Supplemental Digital Content 1, http://links.lww.com/IJG/A890), which may bias the results. Moreover, many patients self-reported race as other or declined to state, which may bias race analyses. Finally, less than half of the VFs included in our study were reliable, which is similar to the reported literature, but may have prompted additional testing and skewed frequency results.

In conclusion, we found that most children with glaucoma had between 1 and 2 VF/year, though close to 40% of patients received <1 VF/year. Compared with children who did not undergo VF testing, those that did were more likely to be older and have better visual acuity. Although no formal guidelines exist regarding recommended VF testing frequency in children with glaucoma, providers should consider barriers to care and future guidelines may require special considerations for children with low vision or different types of glaucoma. Future studies should consider additional sociodemographic and socioeconomic factors as potential barriers to care of VF testing frequency.

## Supplementary Material
