# Supplementary material for: Visual Field Testing Frequency and Associations in Children With Glaucoma
Source: J Glaucoma. 2024 Apr 23;33(7):499–504. doi: 10.1097/IJG.0000000000002406 (PMC11210941; doi:10.1097/IJG.0000000000002406)
Supplement: Supplementary file 1 [file ijg-33-499-s001.docx]

Supplemental Table 2. Multivariable logistic regression for patient baseline characteristics associated with having ≥1 visual field test

|  |  | Adjusted OR  (95% CI) | Adjusted p-value |
| --- | --- | --- | --- |
| **Age (years) at first office visit or first visual field test*** | per 1 year  increase | 1.28  (1.05, 1.56) | **0.01** |
|  |  |  |  |
| **logMAR visual acuity at first office visit or first visual field test**, better eye** | per 0.1 logMAR decrease | 1.27  (1.11, 1.46) | **<0.001** |

OR = odds ratio, CI = confidence interval

*Age at first office visit for those with 0 visual field tests; age at first visual field test for those with ≥1 visual field tests

**logMAR visual acuity at first office visit for those with 0 visual field tests; logMAR visual acuity at first visual field test for those with ≥1 visual fields tests
