# Supplementary material for: Visual Field Testing Frequency and Associations in Children With Glaucoma
Source: J Glaucoma. 2024 Apr 23;33(7):499–504. doi: 10.1097/IJG.0000000000002406 (PMC11210941; doi:10.1097/IJG.0000000000002406)
Supplement: Supplementary file 2 [file ijg-33-499-s002.docx]

Supplemental Table 4. Patient characteristics stratified by visual field testing frequency

|  | **Visual field testing frequency** | | |  |
| --- | --- | --- | --- | --- |
|  | <1 VF/year (N=24) | ≥1 to <2 VFs/year (N=28) | ≥2 VFs/year (N=9) | p-value |
| **Age (years) at the first visual field test** |  |  |  | 0.12 |
| Mean (SD) | 12.4 (3.2) | 11.1 (2.1) | 12.8 (3.2) |  |
| Median (Range) | 11.7 (7.7-18.7) | 11.0 (6.8-14.2) | 12.9 (8.5-16.8) |  |
| Q1, Q3 | 9.9, 15.0 | 9.6, 13.0 | 11.2, 15.3 |  |
|  |  |  |  |  |
| **Self-reported race** |  |  |  | **0.04** |
| White | 4 (16.7%) | 6 (21.4%) | 1 (11.1%) |  |
| Asian | 0 (0.0%) | 6 (21.4%) | 2 (22.2%) |  |
| Black | 5 (20.8%) | 0 (0.0%) | 0 (0.0%) |  |
| Multiracial | 2 (8.3%) | 1 (3.6%) | 0 (0.0%) |  |
| Other or declined to state | 13 (54.2%) | 15 (53.6%) | 6 (66.7%) |  |
|  |  |  |  |  |
| **Ethnicity** |  |  |  | 0.48 |
| Hispanic or Latino | 11 (45.8%) | 11 (39.3%) | 5 (55.6%) |  |
| Not Hispanic or Latino | 11 (45.8%) | 17 (60.7%) | 4 (44.4%) |  |
| Unknown | 2 (8.3%) | 0 (0.0%) | 0 (0.0%) |  |
|  |  |  |  |  |
| **Sex (% male)** |  |  |  | 0.28 |
| Male | 13 (54.2%) | 9 (32.1%) | 4 (44.4%) |  |
|  |  |  |  |  |
| **Primary Language** |  |  |  | 0.19 |
| English | 21 (87.5%) | 20 (71.4%) | 5 (55.6%) |  |
| Spanish | 3 (12.5%) | 5 (17.9%) | 3 (33.3%) |  |
| Other | 0 (0.0%) | 3 (10.7%) | 1 (11.1%) |  |
|  |  |  |  |  |
| **Glaucoma Diagnosis** |  |  |  | 0.84 |
| Primary congenital glaucoma | 6 (25.0%) | 8 (28.6%) | 1 (11.1%) |  |
| Juvenile open-angle glaucoma | 3 (12.5%) | 2 (7.1%) | 0 (0.0%) |  |
| Associated with non-acquired ocular anomalies | 4 (16.7%) | 5 (17.9%) | 2 (22.2%) |  |
| Associated with non-acquired systemic disease | 1 (4.2%) | 2 (7.1%) | 0 (0.0%) |  |
| Associated with acquired conditions | 3 (12.5%) | 6 (21.4%) | 4 (44.4%) |  |
| Glaucoma following cataract surgery | 7 (29.2%) | 5 (17.9%) | 2 (22.2%) |  |
|  |  |  |  |  |
| **Distance to Provider (miles)** |  |  |  | 0.22 |
| 0-25 | 9 (37.5%) | 11 (39.3%) | 4 (44.4%) |  |
| 25-50 | 9 (37.5%) | 5 (17.9%) | 1 (11.1%) |  |
| 50-200 | 3 (12.5%) | 11 (39.3%) | 3 (33.3%) |  |
| > 200 | 3 (12.5%) | 1 (3.6%) | 1 (11.1%) |  |
|  |  |  |  |  |
| **Insurance Type** |  |  |  | 0.46 |
| No insurance | 0 (0.0%) | 1 (3.6%) | 1 (11.1%) |  |
| Public insurance | 16 (66.7%) | 18 (64.3%) | 4 (44.4%) |  |
| Private insurance | 8 (33.3%) | 9 (32.1%) | 4 (44.4%) |  |
|  |  |  |  |  |
| **logMAR visual acuity, better eye** |  |  |  | **0.03** |
| Mean (SD) | 0.4 (0.4) | 0.2 (0.3) | 0.2 (0.4) |  |
| Median (Range) | 0.2 (0.0-1.3) | 0.1 (-0.1-0.9) | 0.1 (-0.1-1.0) |  |
| Q1, Q3 | 0.1, 0.8 | 0.0, 0.2 | 0.0, 0.5 |  |
|  |  |  |  |  |
| **logMAR visual acuity, worse eye** |  |  |  | 0.20 |
| Mean (SD) | 0.7 (0.5) | 0.5 (0.5) | 0.6 (0.5) |  |
| Median (Range) | 0.8 (0.0-1.6) | 0.4 (0.0-1.8) | 0.5 (0.0-1.5) |  |
| Q1, Q3 | 0.2, 1.2 | 0.1, 0.7 | 0.2, 0.8 |  |
|  |  |  |  |  |
| **First VF VFI, better eye (percentage)** |  |  |  | 0.89 |
| Mean (SD) | 90 (20) | 90 (20) | 90 (20) |  |
| Median (Range) | 100 (30-100) | 90 (20-100) | 100 (40-100) |  |
| Q1, Q3 | 90, 100 | 90, 100 | 90, 100 |  |
|  |  |  |  |  |
| **First VF VFI, worse eye (percentage)** |  |  |  | 0.60 |
| Mean (SD) | 80 (20) | 70 (30) | 70 (40) |  |
| Median (Range) | 80 (30-100) | 80 (10-100) | 80 (0-100) |  |
| Q1, Q3 | 70, 100 | 60, 90 | 60, 100 |  |
|  |  |  |  |  |
| **First VF MD, better eye** |  |  |  | 0.84 |
| Mean (SD) | -7.0 (6.9) | -5.9 (5.9) | -6.2 (7.5) |  |
| Median (Range) | -4.8 (-23.5-0.1) | -6.1 (-28.32-4.1) | -5.0 (-23.9-0.2) |  |
| Q1, Q3 | -8.6, -2.3 | -8.0, -1.8 | -6.0, -1.8 |  |
|  |  |  |  |  |
| **First VF MD, worse eye** |  |  |  | 0.90 |
| Mean (SD) | -11.3 (7.3) | -12.0 (8.5) | -12.8 (11.4) |  |
| Median (Range) | -10.7 (-23.5--1.5) | -9.7 (-31.9--0.7) | -9.7 (-32.9-0.2) |  |
| Q1, Q3 | -18.1, -5.4 | -18.9, -6.4 | -19.9, -5.7 |  |
|  |  |  |  |  |
| **VF follow-up length (years)** |  |  |  | **<0.001** |
| Mean (SD) | 3.2 (1.3) | 2.6 (1.5) | 1.0 (1.0) |  |
| Median (Range) | 3.6 (1.0-4.7) | 2.3 (0.6-4.8) | 0.6 (0.2-3.4) |  |
| Q1, Q3 | 1.9, 4.3 | 1.3, 4.2 | 0.45, 0.9 |  |
|  |  |  |  |  |
| **Number of office visits per year** |  |  |  | **<0.001** |
| Mean (SD) | 2.0 (1.5) | 2.5 (1.4) | 4.7 (3.2) |  |
| Median (Range) | 1.4 (0.7-6.9) | 2.2 (0.2-6.2) | 3.0 (1.1-9.4) |  |
| Q1, Q3 | 1.0, 2.2 | 1.7, 2.9 | 2.4, 7.8 |  |
|  |  |  |  |  |
| **Compliance score** |  |  |  | **0.03** |
| Mean (SD) | 0.7 (0.3) | 0.9 (0.1) | 0.9 (0.1) |  |
| Median (Range) | 0.9 (0.0-1.0) | 1.0 (0.5-1.0) | 1.0 (0.6-1.0) |  |
| Q1, Q3 | 0.6, 1.0 | 0.9, 1.0 | 0.8, 1.0 |  |

VF = visual field, VFI = visual field index, MD = mean deviation

Bolded p-values are statistically significant
