# Supplementary material for: Visual Field Testing Frequency and Associations in Children With Glaucoma
Source: J Glaucoma. 2024 Apr 23;33(7):499–504. doi: 10.1097/IJG.0000000000002406 (PMC11210941; doi:10.1097/IJG.0000000000002406)
Supplement: Supplementary file 3 [file ijg-33-499-s003.docx]

Supplemental Table 6. Average compliance score comparisons by self-reported race

|  | Asian (N=8) | White (N=11) | Black  (N=5) | Multiracial  (N=3) | Other or declined to state (N=34) | p-value |
| --- | --- | --- | --- | --- | --- | --- |
| **Compliance score** |  |  |  |  |  | **0.01** |
| Mean (SD) | 0.97 (0.06) | 0.91 (0.14) | 0.45 (0.34) | 0.89 (0.19) | 0.85 (0.21) |  |
| Median (Range) | 1.00 (0.83-1.00) | 1.00 (0.63-1.00) | 0.50 (0.00-0.93) | 1.00 (0.67-1.00) | 0.92 (0.00-1.00) |  |
| Q1, Q3 | 0.94, 1.00 | 0.92, 1.00 | 0.33, 0.50 | 0.67, 1.00 | 0.79, 1.00 |  |
